# Severe Heterotopic Ossification with Proximal Entrapment of the Ulnar Nerve following Primary Anterior Shoulder Dislocation

**DOI:** 10.1155/2020/8883758

**Published:** 2020-10-03

**Authors:** Malik Jessen, Christian Gerhardt, Lars-Johannes Lehmann, Jonas Schmalzl

**Affiliations:** Department of Traumatology and Hand Surgery, St. Vincentius Clinic, ViDia Clinics, Teaching Hospital of the Albert-Ludwigs-University Freiburg, Suedendstraße 32, 76137 Karlsruhe, Germany

## Abstract

Heterotopic ossifications (HO) in the shoulder are rare. The effectiveness of conservative treatment is limited, and therefore, symptomatic cases are usually treated surgically. However, there are no guidelines for the surgical treatment of HO. Herein, we report the case of a 45-year-old man with severe HO and proximal entrapment of the ulnar nerve following primary anterior shoulder dislocation without concomitant injuries (e.g., fracture and rotator cuff tears). Surgical intervention was indicated, including resection of HO and neurolysis of the brachial plexus. Nine months after surgery, the patient presented with restored shoulder function, pain relief, and good patient satisfaction. The case shows that the ulnar nerve can also be impaired due to HO following shoulder dislocation.

## 1. Introduction

Heterotopic ossifications (HO) in the shoulder are rare and can occur after trauma (especially in cases with brain and HDCRO_8883758spinal cord injury) [1–5], previous surgery [6–10], or burn [11]. The underlying causes are poorly understood [3] and are considered as multifactorial. It is suspected that local inflammatory processes are responsible for the migration of mesenchymal stem cells (to the site of injury), whereby cartilage is formed and later remodeled to HO [3]. Besides HO in the shoulder following central nervous system injuries, most of the cases in the literature described HO in the shoulder after arthroplasty [6, 7, 10, 12–14]. HO is often treated conservatively, e.g., with radiation [5], indomethacin, bisphosphonate, or physiotherapy [15]. The effectiveness of conservative treatment is limited, and therefore, symptomatic cases are treated surgically [2, 8] even though there is a higher risk of adverse effects. However, there are no guidelines for the surgical treatment of HO, and decisions are always made on an individual basis as troublesome HO of the shoulder is considered very rare [2]. This case report demonstrates an extensive HO with symptomatic ulnar nerve entrapment. The HO occurred after traumatic primary anterior shoulder dislocation with intact rotator cuff and without fracture, representing a very unusual cause for HO formation.

## 2. Case Presentation

**Figure 1:**
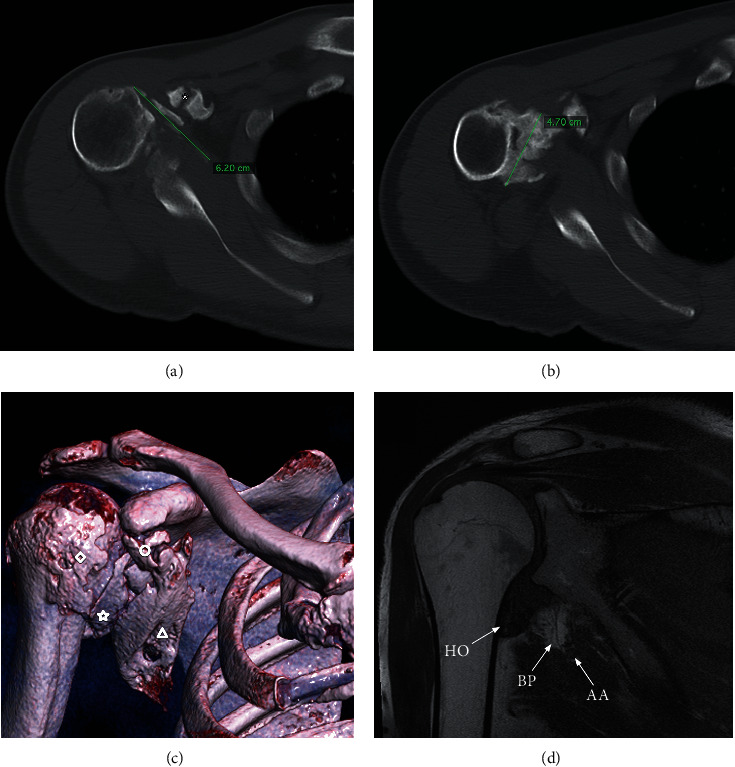
(a–d) 9 months after traumatic anterior dislocation, preoperative CT and MRI images demonstrating severe HO. (a) HO along the bony insertion and ventral side of the subscapularis muscle (green line) and around the conjoint tendons (asterisk) in the axial CT plane. (b) HO extending from the inferior glenoid to the proximal humeral shaft and axillary recess in axial CT plane. (c) 3D CT scan showing the extent of HO, primarily in the ventral part of the subscapularis muscle (triangle), in the axillary recess (asterisk), conjoint tendons (circle), and ventrally of the proximal humerus (diamond). (d) MRI demonstrating close location of the HO (HO) and both axillary artery (AA) and nerves of the brachial plexus (BP).

**Figure 2:**
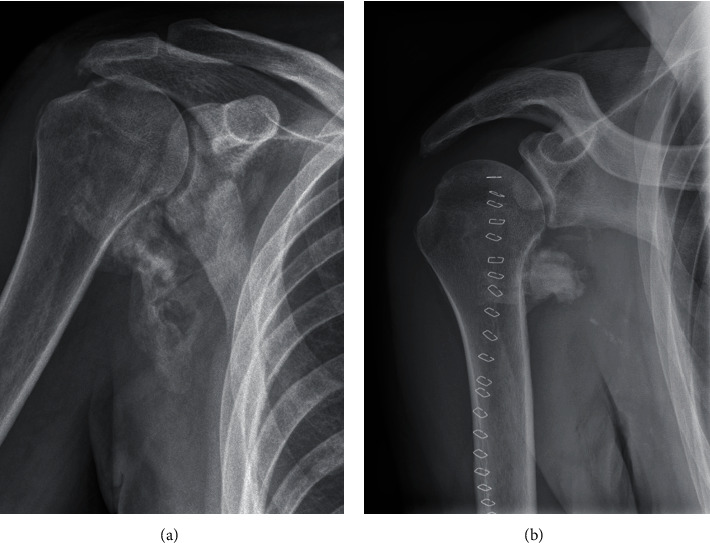
(a, b) AP radiograph demonstrating the extent of the HO one week before (a) and two days after (b) surgery. A small residual of the HO remained (b).

A 45-year-old male, right-dominant, Caucasian patient had a right primary anterior shoulder dislocation after a car accident. Due to burns at the left flank of about 9% of the body surface area caused by the car accident, the patient underwent several skin reconstructions. He had no previous shoulder pathology or other illnesses. The shoulder was reduced in an outside trauma clinic followed by conservative treatment. At 4 months after trauma, the patient presented initially in our institution with posttraumatic stiffness (passive range of motion (ROM): forward flexion 40°, abduction 30°, external rotation 0°), decreased sensation of the fingers 4 and 5, impaired strength with atrophic interosseous muscles, and palpatory distinct, painful ossifications in the axilla, and conjoint tendons. Electroneurography showed evidence for isolated proximal functional disorder of the ulnar nerve with all other nerves intact. We discussed the therapeutic options and first decided to try and exhaust conservative treatment. 9 months after the trauma, the patient presented with deteriorating symptoms and with constantly palpable and painful HO in the axilla and around the conjoint tendons. Plane radiographs and magnet resonance imaging (MRI) showed unaltered HO (Figure 1). The shoulder stiffness had worsened with a fixed internal rotation position of the arm (passive ROM: forward flexion 35°, abduction 35°, external rotation -15°). The impaired sensation of the fingers 4 and 5 as well as the reduced strength of the interosseous muscles remained unaltered. Consequently, a surgical intervention was indicated. Preoperative, radiation was applied for recurrence prophylaxis. Computer tomography (CT) diagnostics revealed pronounced HO throughout the axillary recess, partially encasing neurovascular structures, conjoint tendons, and the subscapularis muscle (Figure 1). Surgical procedure using a deltopectoral and transaxillary approach included resection of HO, neurolysis of the brachial plexus including the axillary artery, tenolysis of the conjoint tendons, and tenotomy of the long head of the biceps. The tendons of the pectoralis major, pectoralis minor, and latissimus dorsi were temporarily detached for complete removal of the HO and neurolysis. The whole ventral side of the subscapularis muscle appeared ossificated, which could explain the fixed internal rotation position of the shoulder. After HO removal (Figure 2), flexion and abduction improved intraoperatively. In addition, external rotation improved from -15° to 40°. Postoperatively, the patient was treated with indomethacin 150 mg for 4 weeks for recurrence prophylaxis and painkillers. Physiotherapy was initiated on the first postoperative day with no restriction in ROM but limited strength for 6 weeks. 9 months after surgery, the patient reported an improvement of both pain (preoperative VAS 7 to postoperative VAS 0) and function (preoperative Constant score 18 points to postoperative Constant score 72 points). Also, neuropathy of the ulnar nerve was regressive with only slight numbness of the fourth finger.

## 3. Discussion

Our case report shows that even primary shoulder dislocation without concomitant injuries (e.g., fracture and rotator cuff tears) could lead to severe HO with proximal encasement of the ulnar nerve. Surgical indication was necessary for the treatment of both the symptomatic, severe HO and nerve entrapment. 9 months after surgery, the patient presented with restored shoulder function, pain relief, and good patient satisfaction, although postoperative X-ray had shown a small residual of the HO (Figure 2).

Currently, the only effective treatment for symptomatic HO is surgical excision [2]. According to the literature, early surgical removal improves functional outcome [16, 17], and timing of surgery is not a risk factor for recurrence [2]. However, this has only been shown for HO following traumatic brain damage and spinal cord injury.

To our knowledge, only one case has been described in the literature in which an entrapment of neurovascular structures occurred after glenohumeral HO as a result of arthroplasty [7]. Due to distinct neurological symptoms, a surgical removal of the HO was offered to the patient, who refused it.

One case report describes a HO following anterior shoulder dislocation associated with a full thickness tear of the supraspinatus and subscapularis muscles in a 70-year-old male [18]. The patient was managed with conservative treatment including nonsteroidal anti-inflammatory drugs, physiotherapy, and orthopedic follow-up.

The authors concluded that HO is a potential complication of anterior shoulder dislocation associated with rotator cuff tear, and arranging routine orthopedic follow-up in all patients with anterior shoulder dislocation is necessary.

The patient in our study showed HO after anterior shoulder dislocation without concomitant injuries and therefore presented with less trauma compared to the case described by Patel et al. [18]. HO was also more severe with entrapment of the ulnar nerve in a much younger patient, and surgery was inevitable to improve functional outcome. Whether the burns or the general trauma caused by the car accident contributed to the formation of HO remains unclear.

The case shows that the ulnar nerve can also be impaired due to HO following shoulder dislocation. This represents a particular aspect after shoulder dislocation, as the most common of the nerves to be affected is the axillary nerve due to its proximity being close to the humeral head.

Therefore, we recommend, as Patel et al. [18], an orthopedic follow-up of the patients with shoulder dislocation including neurological examination to detect consequential damage.

In addition, in case of ongoing pain, X-rays should be included to evaluate not just for osseous abnormalities but also for changes in the soft tissue for an early recognition of HO.

## 4. Conclusions

Traumatic shoulder dislocations, especially in the case of a larger, more general trauma to the individual, must be consistently followed up to detect subsequent damage such as HO at an early stage. In addition to the shoulder's functional examination, a detailed neurological examination of the affected extremity should also be performed. If a severe HO leads to neurological deficits as in the presented patient, surgery is indicated.

## Data Availability

Data is available from the corresponding author on reasonable request.
